# Radiographic and clinical evidence: osteoarthritic knee can change surgical result for lumbar degenerative disease patient undergone surgery for 3-year follow-up: a retrospective comparative clinical study

**DOI:** 10.1186/s12891-020-03755-8

**Published:** 2020-11-12

**Authors:** Yong-Chan Kim, Ki-Tack Kim, Kee-Yong Ha, Joonghyun Ahn, Seungnam Ko, Qiang Luo, Sung-Min Kim, Mingyu Kim, Sunin Yoo

**Affiliations:** Department of Orthopaedic Surgery, College of Medicine, Kyung Hee University, Spine Center, Kyung Hee University Hospital at Gangdong, 892, Dongnam-ro, Gandong-gu, Seoul, 05278 South Korea

**Keywords:** Knee osteoarthritis, Spinal sagittal alignment, Clinical outcome, Posterior instrumentation, Interbody fusion, Compensatory mechanism

## Abstract

**Background:**

There is a paucity of reports clarifying the implication of knee osteoarthritis (OA) on spinal sagittal alignment of patients undergone surgery for lumbar spine. This study aimed to analyze how osteoarthritic knee affects radiographic and clinical results of degenerative lumbar disease patients undergone lumbar fusion.

**Methods:**

We retrospectively reviewed the medical records and radiographs of 74 consecutive degenerative lumbar disease patients who underwent posterior instrumentation and fusion surgery between May 2016 and June 2017 and were followed up for minimum 3 years postoperatively. The patients were divided into 2 groups according to the severity of knee OA by Kellgren-Lawrence grading (KLG) scale (group I, KLG 1 or 2 [*n* = 39]; group II, KLG 3 or 4 [*n* = 35]). Patient demographic data, comorbidities, spinal sagittal parameters and clinical scores were extracted and compared at preoperative, postoperative 1 month and the ultimate follow-up between the groups. In radiographic assessment, sagittal alignment parameters and sagittal balance were used. In clinical assessment, the scores of Oswestry disability index (ODI) and Scoliosis Research Society questionnaire (SRS-22) were used. For the frequency analysis of categorical variables across the groups, chi-square test was used and student *t* tests was used to compare the differences of continuous variables.

**Results:**

In radiographic assessment, TLK (thoracolumbar kyphosis), LL (lumbar lordosis), PT (pelvic tilt), C7 SVA (sagittal vertical axis) in both groups improved significantly after surgery (*p* <  0.05). However, LL, PT, C7SVA improved at postoperative 1 month in the group II were not maintained at the ultimate postoperative follow-up. In clinical assessment, preoperative Oswestry disability index (ODI, %) and all SRS-22 subscores of the group I and II were not different (*p* > 0.05). There were significant differences between the groups at the ultimate follow-up in ODI (− 25.6 vs − 12.1, *p* <  0.001), SRS total score (%) (28 vs 20, *p* = 0.037), function subscore (1.4 vs 0.7, *p* = 0.016), and satisfaction subscore (1.6 vs 0.6, *p* < 0.001).

**Conclusion:**

Osteoarthritic knee with KLG 3 or 4 have a negative influence on maintaining postoperative spinal sagittal alignment, balance, and the clinical outcomes achieved immediately by posterior instrumentation and fusion for lumbar degenerative disease.

**Trial registration:**

This study was retrospectively registered with approval by the institutional review board (IRB) of our institution (approval number: 2018–11-007).

## Background

Degeneration of spine and arthritis of knee may be a natural course in ambulating upright human body and the number of patients with problems in both spine and knee is increasing with rising life expectancy in many countries [1]. Many aged patients who undergo spinal surgery frequently present symptoms relating to knee osteoarthritis (OA). Therefore, failure to recognize a concurrent disease may lead to misdiagnosis and possibly erroneous treatment. Although it is difficult to decide what might be the main pathology in order of priority, we sometimes observe the improved spinal alignment or symptoms after elimination of knee problem by total knee arthroplasty [2].

Although there are still no definite studies investigating knee joints’ effect on spinopelvic alignment, Tsuji et al. [3] introduced the knee-spine syndrome: phenomenon of thigh muscle tightness and knee flexion leading decreased lumbar lordosis and sacral inclination while standing in elderly Japanese. And also, Takemitsu et al. [4] previously reported that patients of lumbar degenerative kyphosis with dorsal tilted sacrum were standing in knee-flexion position to gain mechanical advantages. Because motion of the knee joint has such a significant impact on the biomechanics of sagittal balance, it is also required to understand the effect of knee OA on the outcomes of patients who undergo spinal fusion surgery.

To our knowledge, there is a paucity of reports clarifying the implication of knee osteoarthritis on spinal sagittal alignment of patients undergoing posterior instrumentation and fusion. The purpose of this study is thus to evaluate how osteoarthritic knee affects spinal sagittal alignment of degenerative lumbar disease patients undergoing posterior instrumentation and fusion using time dependent outcome analysis.

## Methods

### Study design & patient population

After obtaining informed consent from each patient and approval by the institutional review board (IRB) of our institution (approval number: 2018–11-007), a retrospective study was performed on 74 consecutive patients between the age of 55 to 75 with degenerative lumbar disease treated by decompression and instrumented interbody fusion between May 2016 and June 2017 at a single institution. In this study, we included patients who underwent 1- or 2-level instrumented lumbar interbody fusion with at least 3 years’ postoperative follow-up and with preoperative anteroposterior (AP) standing radiographs of knees. The exclusion criteria of etiology were fracture, tumor, infection, deformity, metabolic or neuromuscular spinal pathology. Also, those with previous history of spinal fusion, knee arthroplasty, leg length discrepancy and additional knee arthroplasty surgery after spinal fusion surgery during the postoperative follow-up were excluded. All patients had persistent or frequently recurrent low back pain with leg pain and were resistant to active non-operative treatment (medications, exercise, injection and bracing) prior to surgery. The patients were divided into two groups by the severity of knee OA in Kellgren-Lawrence Grading (KLG) Scale [5] (Table 1). Group I was made up of the patients of KLG 1 or 2 (*n* = 39) and Group II was made up of the patients of KLG 3 or 4 (*n* = 35). We collected and analyzed patient factors (age at surgery, sex, body mass index (BMI), other medical comorbidities), radiographic parameters and clinical scores.

**Table 1:** Kellgren-Lawrence Grading (KLG) Scale

**Grade 1**	Doubtful narrowing of joint space and possible osteophytic lipping
**Grade 2**	Definite osteophytes, definite narrowing of joint space
**Grade 3**	Moderate multiple osteophytes, definite narrowing of joints space, some sclerosis and possible deformity of bone contour
**Grade 4**	Large osteophytes, marked narrowing of joint space, severe sclerosis and definite deformity of bone contour

### Radiographic assessment

All patients were assessed by 36-in. whole spine standing lateral radiograph preoperatively, at 1-month postoperatively, and at the ultimate follow-up. And also, preoperative standing teleradiographs of lower extremities were taken. Before each session of taking radiographs, the patients were instructed to stand in a comfortable position with the fist-on-clavicle position [6].

**Fig. 1:**
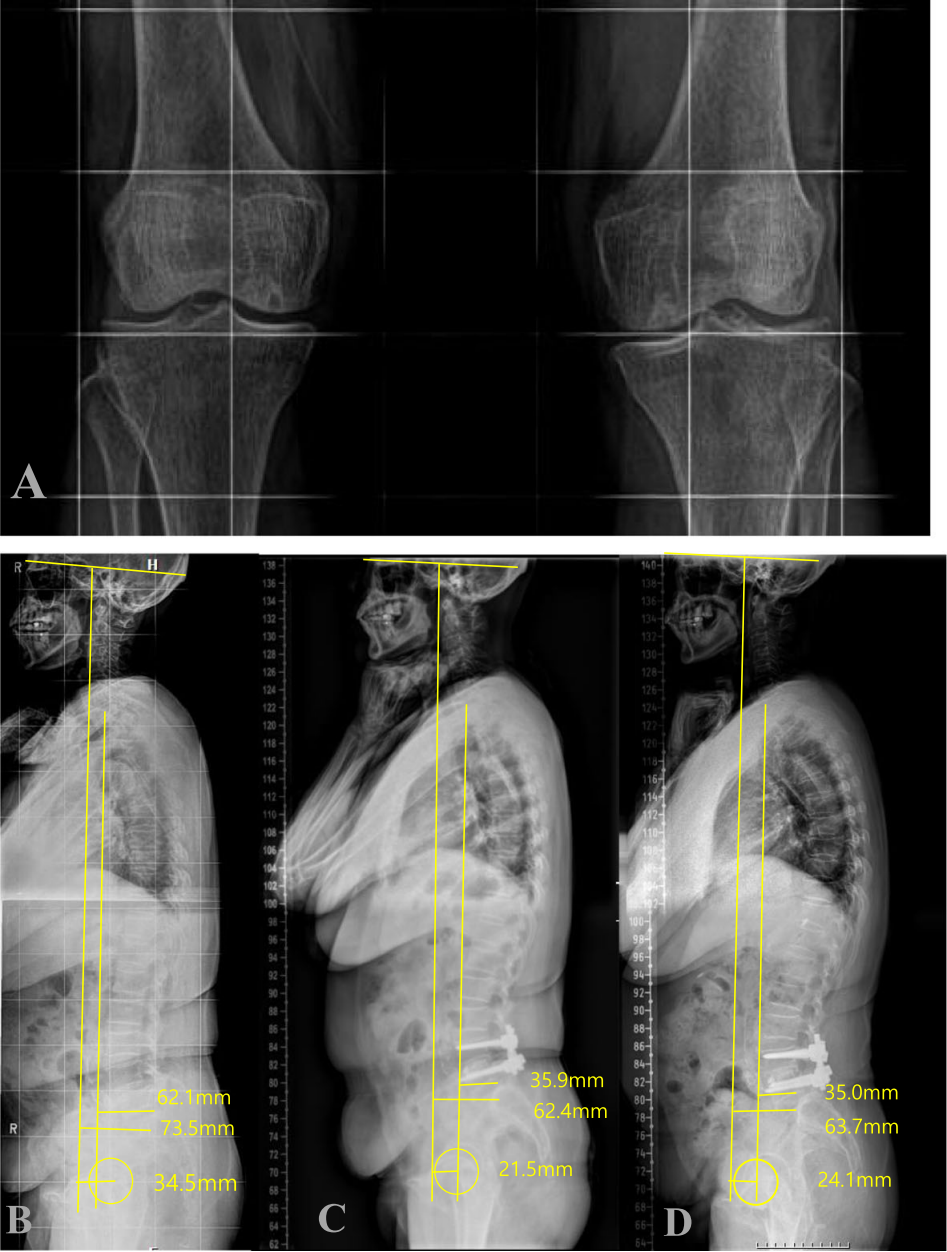
A case of 64-year old female, who underwent L4-L5 instrumented fusion. **a** Preoperative standing knee AP radiograph shows mild (KLG 1) knee OA. Whole spine standing lateral radiographs taken at **b** the preoperative, **c** the postoperative 1 month, and **d** the postoperative 3-year follow-up show the stable maintenance of postoperatively achieved improvement in radiographic sagittal balance

**Fig. 2:**
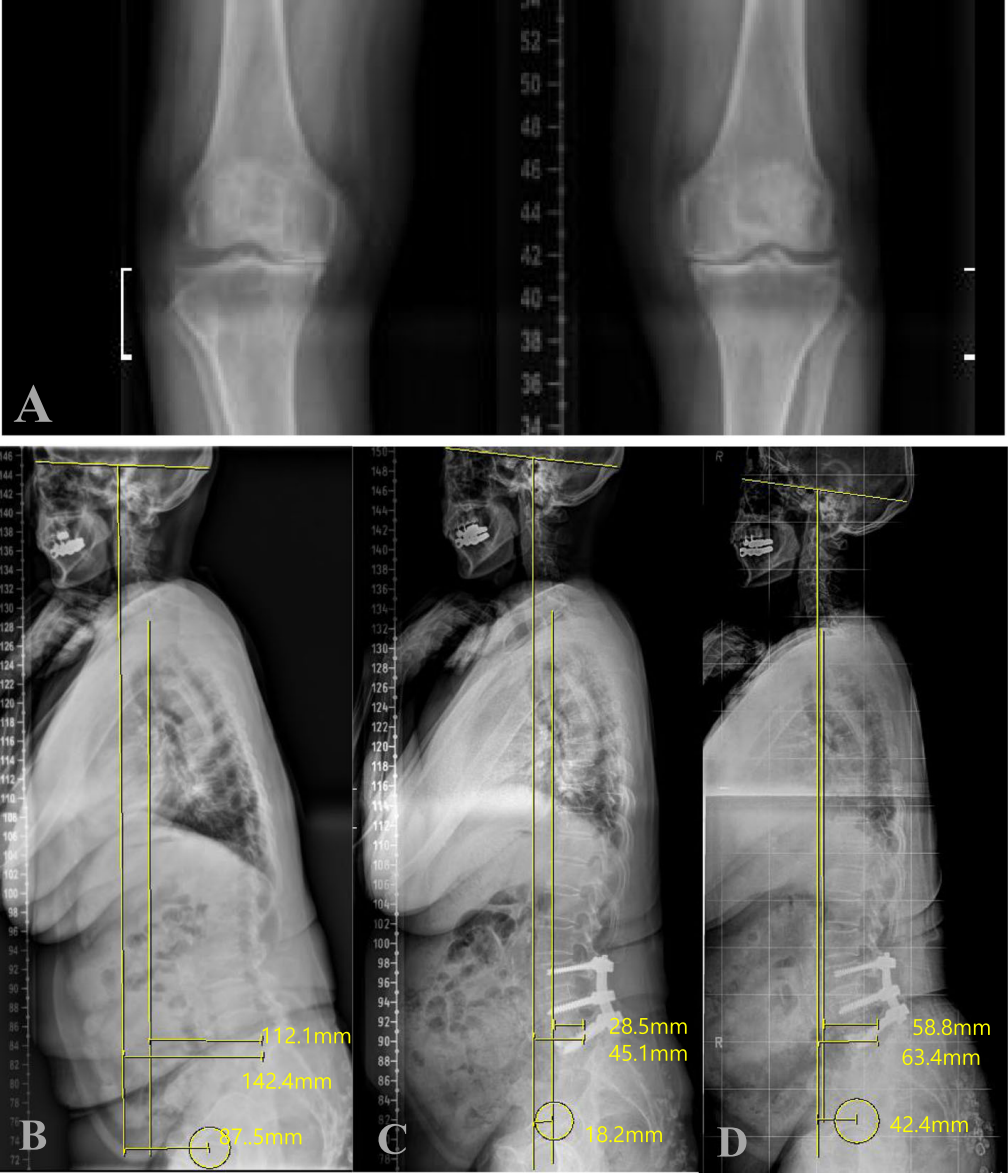
A case of 67-year old female patient, who underwent L3-L5 instrumented fusion. **a** Preoperative standing knee AP radiograph shows severe (KLG 4) knee OA. Whole spine stnading lateral radiographs taken at **b** the preoperative, **c** the postoperative 1 month, and **d** the postoperative 3-year follow-up show reaggravation of radiographic sagittal balance which was improved at postoperative 1 month

The radiographic parameters included (1) C7 sagittal vertical axis(C7SVA); the distance between the vertical plumb line centered in the middle of C7 vertebral body and the posterosuperior corner of S1, (2) Cranial-sagittal vertical axis-hip (CrSVA-H); the distance between the vertical plumb line from the cranial center of gravity (CCG) to the centers of femoral heads [7], (3) Cranial-sagittal vertical axis-Sacrum (CrSVA-S); the distance between the vertical plumb line from the CCG to the posterosuperior corner of S1[7, 4) Cervical lordosis (CL); the angle created by a line parallel to the inferior endplate of C2 and a line parallel to the inferior endplate of C7, (5) Thoracic kyphosis (TK); the angle created by a line parallel to the inferior endplate of T5 and a line parallel to the inferior endplate of T12, (6) Thoracolumbar kyphosis (TLK); the angle created by a line parallel to the superior endplate of T10 and a line parallel to the inferior endplate of L2, (7) Lumbar lordosis (LL); the angle created by a line parallel to the superior endplate of L1 and a line parallel to the endplate of S1, (8) Pelvic tilt (PT); the angle between the vertical line connecting the midpoint of the sacral plate to the femoral head axis and the vertical axis. For all sagittal measurements, the angle was defined to be negative if the curve was lordotic and positive if the curve was kyphotic. The methods used for measurement of parameters representing sagittal vertical axis (C7SVA, CrSVA-H and CrSVA-S) are shown in Figs. 1 and 2 [7].

The CCG was defined as a point approximately 1 cm above and anterior to the external auditory canal, which was nearly equal to the midpoints of the nasion-inion line (root of the nose to the outer cortex of the external occipital protuberance) [8]. All radiographic parameters were measured by PiViewSTAR Software. The measurement was performed by blinded method of two orthopedic spine surgeons (YCK, JA) using the software. Before performing the measurements, each spine surgeon was trained on the use of software and the measurement technique. Each spine surgeon was equally assigned all patients’ radiographs. They performed this process twice and were allowed to complete the measurement at his own pace over the course of 3 weeks. We adopted the mean value of the measurement by each observer.

### Clinical assessment

To evaluate clinical outcomes, we reviewed and used the Oswestry disability index (ODI) scores, Scoliosis Research Society (SRS-22) questionnaire at the preoperative, the postoperative 1 month and the ultimate follow-up.

### Statistical analysis

SPSS package software (IBM SPSS Statistics for Windows, Version 22.0, Armonk, NY; IBM Corp.) was used for statistical analysis. Distribution normality was assessed using the Kolmogorov-Smirnova test. For the frequency analysis of categorical variables across the groups, chi-square test was used and student *t* tests was used to assess differences of continuous measures, according to whether the data followed normal distribution. For most variables for data which were collected before and after surgery, paired *t* test was used to determine whether there was a significant change between the values at the time points. A *p* value < 0.05 was considered statistically significant.

## Results

### Demographics and clinical details

The study population demographics and clinical details for the 74 patients are shown in Table 2.

**Table 2:** Demographics and clinical details of this study

		Knee Osteoarthritis		
	Total cases	Group I (KL 1,2)	Group II (KL 3,4)	*p*-value
n(%)	74 (100)	39 (52.7)	35 (47.3)	
Sex, n (%)				
Male	20 (27.0)	14 (35.9)	6 (17.14)	0.070 ^a^
Female	54 (73.0)	25 (64.1)	29 (82.86)	
Age (mean ± SD), year(s)	67.84 ± 9.38	66.38 ± 10.01	69.46 ± 8.47	0.139^b^
Age > 70 y, n (%)	58 (78.38)	28 (71.79)	30 (85.71)	0.146^a^
BMI (SD), kg/m^2^	25.3 ± 8.9	24.6 ± 5.7	25.9 ± 11.2	0.231^b^
Diagnosis				
Spinal stenosis	35	16	19	0.928^a^
Spondylolisthesis	19	10	9	
Postoperative state of HNP	11	5	6	
Postoperative state of ST	9	5	4	
DM, n (%)	21 (28.38)	12 (30.77)	9 (25.71)	0.630^a^
HTN, n (%)	24 (32.43)	14 (35.90)	10 (28.57)	0.501^a^
Coronary A dis., n (%)	11 (14.86)	7 (17.95)	4 (11.43)	0.431^a^

Values are presented as mean ± standard deviation

^a^ chi-square test

^b^ student t test

*KL* Kellgren-Lawrence grade, *SD* standard deviation, *BMI* Body mass index, *DM* Diabetes mellitus, *HTN* Hypertension, *Coronary A dis*. Coronary artery disease

The mean age of patients was 70.4 years in group I and 69.3 years in group II (*p* = 0.139). There were 14 (35.90%) males in group I and 6 (17.14%) in group II (*p* = 0.07). The body mass index (BMI) score was not significantly different in group II (25.9 kg/m^2^) and in group I (24.6 kg/m^2^) (*p* = 0.231). There were no differences between the groups in preoperative diagnosis. There were no significant differences in medical comorbidity of diabetes (*p* = 0.630), hypertension (*p* = 0.501), coronary artery disease (*p* = 0.431) between the 2 groups.

### Change of radiographic sagittal alignment parameters

The radiographic measurements of sagittal alignment parameters were summarized in Table 3.

**Table 3:** Comparison of radiographic sagittal alignment parameters

	Group I			Group II			Comparison
	Value, mean±S.D.	*p* Preop Versus PO#1 M	*P* PO#1 M Versus Ultimate PO	Value, mean±S.D.	*p* Preop Versus PO#1 M	*P* PO#1 M Versus Ultimate PO	*P*^a^ Group 1 Versus Group 2
CL(°)							
Preop	−14.3 ± 6.8			−12.9 ± 10.8			0.451
PO#1 M	−9.1 ± 7.6	0.043*		−9.3 ± 11.0	0.139		0.584
Ultimate PO	−9.0 ± 7.8		0.744	−10.8 ± 9.7		0.326	0.244
Ultimate PO-Preop	5.3 ± 4.7			2.1 ± 1.8			0.159
Ultimate PO-PO#1 M	0.1 ± 1.3			−1.5 ± 0.9			0.665
TK(°)							
Preop	19.5 ± 12.3			20.7 ± 11.5			0.429
PO#1 M	23.1 ± 9.7	0.122		21.3 ± 11.4	0.774		0.325
Ultimate PO	24.8 ± 10.3		0.395	20.9 ± 13.6		0.620	0.174
Ultimate PO-Preop	5.3 ± 4.2			0.2 ± 8.52			0.203
Ultimate PO-PO#1 M	1.7 ± 0.5			−0.4 ± 1.2			0.599
TLK(°)							
Preop	3.7 ± 2.1			4.3 ± 1.8			0.578
PO#1 M	0.8 ± 1.5	0.029*		1.9 ± 3.3	0.021*		0.438
Ultimate PO	1.2 ± 0.9		0.182	3.8 ± 5.6		0.160	0.306
Ultimate PO -Preop	−2.5 ± 1.1			−0.5 ± 1.6			0.652
Ultimate PO-PO#1 M	0.4 ± 0.9			1.9 ± 1.2			0.744
LL(°)							
Preop	−31.1± 11.3			−29.8 ± 10.2			0.437
PO#1 M	−44.8 ± 8.6	< 0.001*		−42.3 ± 9.3	< 0.001*		0.521
Ultimate PO	−43.4± 10.2		0.782	−35.9 ± 9.2		0.041*	0.015*
Ultimate PO -Preop	−12.3 ± 7.4			−6.1 ± 9.1			0.031*
Ultimate PO-PO#1 M	1.4 ± 1.3			6.4 ± 3.2			0.042*
PT(°)							
Preop	26.8 ± 11.6			24.7 ± 8.4			0.449
PO#1 M	18.5 ± 10.7	< 0.001*		20.5 ± 11.2	0.021*		0.332
Ultimate PO	19.1 ± 7.3		0.861	24.3 ± 10.3		0.019*	0.185
Ultimate PO-Preop	−7.7 ± 5.3			−5.4 ± 3.8			0.223
Ultimate PO-PO#1 M	0.6 ± 0.3			−1.2 ± 2.1			0.095

*Statistically significant if P < 0.05. ^a^*Student t test*

*Preop* Preoperative, *PO#1 M* Postoperative 1 month, *Ultimate PO* The ultimate follow-up, *CL* Cervical lordosis, *TK* Thoracic kyphosis, *TLK* Thoracolumbar kyphosis, *LL* Lumbar lordosis, *PT* Pelvic tilt

In group I, parameters such as CL, TLK, LL, PT between preoperative and postoperative 1 month values showed improvement at postoperative 1 month and the results were maintained until the ultimate follow-up.

In group II, compared to preoperative parameters, parameters at postoperative 1 month values were as follows showing significant improvement; TLK (4.3 vs 1.9, *p* = 0.021), LL (− 29.8 vs − 42.3, *p* < 0.001) and PT (24.7 vs 20.5, p = 0.021), respectively. Among the improved parameters at postoperative 1 month, these results were not maintained and some parameters were significantly deteriorated at the ultimate follow-up; TLK (1.9 vs 3.8, *p* = 0.160), LL (− 42.3 vs − 35.9, *p* = 0.041) and PT (20.5 vs 24.3, *p* = 0.019).

In comparison between group I and II, both postoperative radiographic values at postoperative 1 month were not significantly different in TLK, LL, PT(*p* > 0.05). At the ultimate follow-up, significant differences were found in TLK (1.2 vs 3.8, *p* = 0.022), LL (− 43.4 vs − 35.9, *p* = 0.015), respectively. Moreover, the amount of changes in LL between the preoperative and the ultimate follow-up (− 12.3 vs − 3.1, *p* = 0.031) and between the postoperative 1 month and the ultimate follow-up (1.4 vs 6.4, *p* = 0.042) were also significantly different.

### Change of radiographic sagittal balance parameters

The radiographic measurements of sagittal balance parameters were summarized in Table 4.

**Table 4:** Comparison of radiographic sagittal balance parameters

	Group I			Group II			Comparison
	Value, mean ± S.D.	*P* Versus Preop	*P* PO#2 M Versus Ultimate PO	Value, mean ± S.D.	*p* Versus Preop	*P* PO#2 M Versus Ultimate PO	*P*^a^ Group 1 Versus Group 2
C7SVA (mm)							
Preop	39.2 ± 15.8			47.3 ± 9.3			0.234
PO#1 M	23.2 ± 19.9	< 0.001*		28.3 ± 11.5	< 0.001*		0.125
Ultimate PO	22.1 ± 14.6	< 0.001*	0.188	43.7± 5.9	0.043	< 0.001*	< 0.001*
Ultimate PO-Preop	-17.1 ± 19.2			-3. ± 2.6			< 0.001*
Ultimate PO-PO#1 M	-1. ± 0.6			15. ± 9.5			< 0.001*
CrSVA-S (mm)							
Preop	50.2 ± 18.7			48.9 ± 17.8			0.249
PO#1 M	31.5 ± 18.4	< 0.001*		33.5 ± 15.3	< 0.001*		0.333
Ultimate PO	33.2 ± 11.6	< 0.001*	0.474	45.5 ± 23.4	0.188	< 0.001*	< 0.001*
Ultimate PO-Preop	-17.0 ± 8.6			-3.4 ± 2.6			0.021*
Ultimate PO-PO#1 M	1.7 ± 1.2			12 ± 5.5			< 0.001*
CrSVA-H (mm)							
Preop	−19.1 ± 6.1			−18.0 ± 9.3			0.519
PO#1 M	−8.5 ± 6.5	< 0.001*		−9.6 ± 6.9	< 0.001*		0.425
Ultimate PO	−7.2 ± 3.5	< 0.001*	0.112	−16.6 ± 7.3	0.103	0.008*	< 0.001*
Ultimate PO-Preop	11.9 ± 8.9			1.4 ± 0.9			< 0.001*
Ultimate PO-PO#1 M	1.3 ± 0.6			-7.0 ± 4.3			0.022*

*Statistically significant if p < 0.05. ^a^*Student t test*

*Preop* Preoperative, *PO#1 M* Postoperative 1 month, *Ultimate PO* The ultimate follow-up, *C7SVA* C7 sagittal vertical axis, *CrSVA-H* Head sagittal vertical axis-Hip, *CrSVA-S* Head sagittal vertical axis-Sacrum;

In group I, between the preoperative and the postoperative 1 month values, all parameters showed improvement such as C7SVA (39.2 vs 23.2, *p* < 0.001), CrSVA-S (50.2 vs 31.5, *p* < 0.001), CrSVA-H (− 19.1 vs − 8.5, *p* < 0.001), respectively. The immediate improvement achieved at 1 month were maintained in all parameters until the ultimate follow-up.

In group II, compared to preoperative parameters, parameters at postoperative 1 month values were as follows showing significant improvement; C7SVA (47.3 vs 28.3, *p* < 0.001), CrSVA-S (48.9 vs 33.5, p < 0.001), CrSVA-H (− 18.0 vs − 9.6, p < 0.001), respectively. In all 3 parameters, the improvement at postoperative 1 month were significantly deteriorated at the ultimate follow-up; C7SVA (28.3 vs 47.3, p < 0.001), CrSVA-S (33.5 vs 45.5, *p* < 0.001), CrSVA-H (− 9.6 vs − 16.6, *p* = 0.008).

Between the group I and II, both postoperative radiographic values at postoperative 1 month were not significantly different in C7SVA (23.2 vs 28.3, *p* = 0.125), CrSVA-S (31.5 vs 33.5, *p* = 0.333), CrSVA-H (− 8.5 vs − 9.6, *p* = 0.425), respectively. At the ultimate follow-up, significant differences were found in C7SVA (22.1 vs 43.7, *p* < 0.001), CrSVA-S (33.2 vs 45.5, p < 0.001), CrSVA-H (− 7.2 vs − 16.6, p < 0.001), respectively. Furthermore, in terms of amount of changes, there were significant differences between the preoperative and the ultimate follow-up in C7SVA (− 17.1 vs − 3.6. *p* < 0.001), CrSVA-S (− 17.0 vs − 3.4, *p* = 0.021) and CrSVA-H (11.9 vs 1.4, p < 0.001). Also, there were significant differences in amount of the changes between the postoperative 1 month and the ultimate follow-up in C7SVA (− 1.1 vs 15.4, *p* < 0.001), CrSVA-S (1.7 vs 12, *p* < 0.001) and CrSVA-H (1.3 vs − 7.0, *p* = 0.022).

### The changes in scores of clinical parameters

The clinical results were summarized in Table 5.

**Table 5:** Changes of clinical scores between the Groups

	Group I		Group II		Comparison
	Value mean ± SD	p Preop vs Ultimate PO	Value mean ± SD	p Preop vs Ultimate PO	p Group I vs II
ODI score (100%)					
Preop	47.1 ± 21.5		45.0 ± 22.6		0.501
Ultimate PO	21.5 ± 13.1	< 0.001*	32.9 ± 12.2	0.011*	< 0.001*
Ultimate PO-Preop	−25.6 ± 13.7		−12.1 ± 11.6		< 0.001*
SRS total score (100%)					
Preop	50.8 ± 20.4		47.6 ± 20.8		0.245
Ultimate PO	78.8 ± 20.4	0.005*	67.6 ± 16.4	0.268	0.111
Ultimate PO-Preop	28 ± 25.6		20 ± 14.8		0.037*
SRS Pain (5)					
Preop	1.7 ± 0.9		1.4 ± 1.2		0.545
Ultimate PO	3.6 ± 0.3	< 0.001*	3.1 ± 0.9	< 0.001*	0.395
Ultimate PO-Preop	1.9 ± 0.9		1.7 ± 0.8		0.509
SRS Self-image (5)					
Preop	2.9 ± 0.8		2.8 ± 1.1		0.741
Ultimate PO	4.2 ± 1.1	< 0.001*	3.9 ± 0.8	0.042*	0.397
Ultimate PO-Preop	1.3 ± 0.7		1.1 ± 0.7		0.525
SRS Function (5)					
Preop	2.5 ± 0.8		2.6 ± 0.9		0.774
Ultimate PO	3.9 ± 1.0	< 0.001*	3.3 ± 0.7	0.044*	0.123
Ultimate PO - Pre-op	1.4 ± 0.7		0.7 ± 0.6		0.016*
SRS Satisfaction (5)					
Preop	2.6 ± 1.5		2.4 ± 1.1		0.622
Ultimate PO	4.2 ± 1.7	< 0.001*	3.0 ± 1.1	0.161	< 0.001*
Ultimate PO-Preop	1.6 ± 2.8		0.6 ± 1.0		< 0.001*
SRS Mental health (5)					
Preop	3 ± 1.1		2.7 ± 0.9		0.391
Ultimate PO	3.8 ± 1.0	0.221	3.6 ± 0.6	0.391	0.624
Ultimate PO-Preop	0.8 ± 1.3		0.9 ± 0.6		0.777

* Statistically significant if p < 0.05 in student t test

Preop Preoperative, *PO* Postoperative follow-up, *SD* Standard deviation, *ODI* Oswestry disability index questionnaire, *SRS* Scoliosis Research Society-22 questionnaire

Clinical parameters compared between the preoperative and the ultimate values in group I showed significant improvement in ODI (%) (47.1 vs 21.5, *p* < 0.001), SRS total score (%) (50.8 vs 78.8, *p* = 0.005), pain (1.7 vs 3.6, p < 0.001), self-image (2.9 vs 4.2, p < 0.001), function (2.5 vs 3.9, p < 0.001), and satisfaction (2.6 vs 4.2, p < 0.001), respectively.

Clinical parameters compared between the preoperative and the ultimate values in group II showed significant improvement in ODI (%) (45.0 vs 32.9, *p* = 0.011), SRS subscore of pain (1.4 vs 3.1, *p* < 0.001), self-image (2.8 vs 3.9, *p* = 0.042) and function (2.6 vs 3.3, *p* = 0.044), respectively.

In comparison of scores of clinical parameters between the groups, it was not different in preoperative ODI (%) (47.1 vs 45.0, *p* = 0.501). ODI at the ultimate follow-up compared to the preoperative improved in each group (21.5, *p* < 0.001; 32.9, *p* < 0.011). Between the groups, there were significant differences in ODI at the ultimate follow-up (21.5 vs 32.9, *p* < 0.001) and the amount of improved ODI (− 25.6 vs − 12.1, p < 0.001), showing superiority in group I. Regarding SRS-22 scores, there were no significant differences between the groups preoperatively. At the ultimate follow-up, however, values in group I was higher in SRS total score (%) (78.8 vs 67.6, *p* = 0.037), and satisfaction (4.2 vs 3.0, *p* < 0.001). The amount of improvement in group I was higher in SRS score of total (%) (28 vs 20, p = 0.037), function (1.4 vs 0.7, *p* < 0.016), and satisfaction (1.6 vs 0.6, p < 0.001).

## Discussion

Ambulatory humans should maintain upright standing position with well-aligned weight bearing segments to achieve the minimization of energy expenditure [9, 10]. Not to mention, the sagittal spinal alignment is crucial in maintaining well-aligned standing position. However, if any pathological or degenerative changes in the spine, pelvis or lower extremities occurs, they would disrupt the interactive balanced posture; then, the spine–pelvis–leg alignment should be restored by compensatory changes in other segments. Sagittal spinopelvic alignment and compensatory mechanisms in patients with spinal disorders were reported regarding a relationship between pelvis and spine, and it has been well known that spinal sagittal imbalance leads to adaptive changes in the pelvis, hip joint through compensatory mechanism [2, 11–14]. And also, knee flexion [15] is also well-known compensatory mechanism accompanied by ankle extension (dorsiflexion) in sagittal plane. Aside from compensation of spine itself, therefore, postoperative symptoms arising from the lumbar spine might be insufficient compensatory mechanism caused indirectly by hip [16, 17] and knee osteoarthritis (OA) [6, 18], et cetera. Knee OA can be easily assessed in coronal plane radiographs such as standing anteroposterior or 45-degree flexed posteroanterior (Rosenberg’s) view and we used the former method.

In the current study, TLK, LL and PT in both groups improved at postoperative 1 month. Whereas those results achieved at 1 month in group I were maintained until the ultimate follow-up, LL and PT in group II showed deterioration at the ultimate follow-up. Particularly, the interval changes in LL from the preoperative to the ultimate follow-up and from postoperative 1 month to the ultimate follow-up were significantly different between the groups showing inferior outcome in group II. This is considered postoperative LL was more closely and directly associated with severe knee OA than other spinopelvic parameters. It is keeping with the previous literatures that the knee OA with flexion contracture can lead to decreased lumbar lordosis [2, 18] and PT will not be directly correlated to LL if lumbar spine is flexible [2]. In normal compensation mechanism, postoperatively restored LL should have result in decreased PT and decreased knee flexion. However, in severe knee OA such as group II, we think this mechanism does not seem to work normally. The phenomenon can be explained by the literatures investigating biomechanical incapability of OA knee [9, 19]. Messier et al. [19] suggested that patients with knee OA reduce the knee extension moments and Astephen et al. [9] also reported decreased early stance knee extension moments of progressive OA in biomechanical analysis related with knee OA severity. On the correlation of knee and LL, Murata et al. [18] reported significantly reduced LL in patients whose limitation of knee extension was more than 5 degrees. And Lee et al. [2] also reported decreased PT and increased sacral slope after total knee arthroplasty in patients with preoperative knee flexion contracture more than 10 degrees. Presumably, although restored LL after spinal surgery allow the margin of compensation to tilted pelvis and flexed knees, flexion contracture of OA knee will prohibit knee extension, subsequent limitation of motion in hip and ankle joint.

However, CL and TK were not different between the groups postoperatively. We think cervical and thoracic spine is located so distant from the knee joints compared to lumbar spine that they cannot be easily affected by knee OA. Another reason to explain this is the compensatory changes in TK cannot work well in older patients because thoracic hypokyphosis requires strong muscle tone, which maybe deficient in older patients [20]. Also, it is because cervical lordosis in older patients is usually considered stiff and already recruited to maintain horizontal gaze [20].

Regarding global sagittal balance, C7SVA, CrSVA-H and CrSVA-H improved at postoperative 1 month showed aggravation at the ultimate follow-up in group II. Despite the aggravation in group II until the ultimate follow-up, C7SVA was still significantly different between the preoperative and the ultimate follow-up. But we found no significant differences between the preoperative value and the ultimate follow-up value in CrSVA-S and CrSVA-H, which means C7SVA is less sensitive than CrSVA-S and CrSVA-H in detecting mild deterioration of global sagittal balance. It was also reported CrSVA was more correlated with ODI and all SRS subscores than C7SVA [7], which could not consider the motion of cervical spine [21]. But we still think further investigation of cranial parameters is warranted to clarify their meaning as global alignment parameters.

However, even in patients with insufficient sagittal correction after spinal fusion surgery, postoperative sagittal imbalance often improves with time [22]. Although it is not always clearly understood, it can be sometimes explained by the improvement of pain and increased function after laminectomy facilitating patients’ restoration of upright posture [12]. In our study, the preoperative score of SRS pain and function significantly improved in both groups at the ultimate follow-up. However, group I showed significantly better outcomes than group II in comparison of the amount of improved ODI score (21.5 vs 32.9, *p* < 0.001), SRS total (%) (78.8 vs 67.6, *p* = 0.037), improved SRS function (1.4 vs 0.7, *p* = 0.016), and satisfaction (4.2 vs 3.0, p < 0.001). Moreover, although group II showed improvement at the ultimate follow-up in ODI, SRS pain, self-image and function scores, their satisfaction and mental health score were not improved compared to the preoperative. These results indicate that clinical prognosis after spinal fusion surgery may be unfavorable if severe knee OA in patients exists concurrently. According to the report of Ho et al. [23], at postoperative 1 year, ODI scores were shown to be affected by the operational level, the preoperative ODI, and the presence of advanced radiographic knee OA (Kellgren/Lawrence grades III and IV) (*P* < 0.05). This phenomenon was also supported in the study of the relationship between clinical outcomes and sagittal radiographic parameters by Lafage et al. [24]. Therefore, when spine surgeons plan spinal instrumented fusion surgery in patients with osteoarthritic knees, the severity of osteoarthritic knee should be evaluated preoperatively and simultaneous treatment of knee OA should be considered in patients who are required to undergo spinal instrumented fusion surgery.

In summary, regardless of the severity in preoperative knee OA, the initial improvement in radiographic measurement and clinical outcomes can be achieved by spinal surgery not only with instrumented correction of spinal alignment but also with the spinal decompression. However, patients with knee OA of K-L grade 3 or 4 were lacking in compensatory mechanism by knee joint motion maintaining immediate postoperative spinal sagittal balance. The most important mechanism we explained was accompanying knee flexion contracture in OA knee which had less capacity of extensive joint motion to accommodate improved spinal balance.

We acknowledge that this study has several limitations. To elucidate the effect of the knee OA on maintaining the immediate postoperative outcomes of spinal surgery, whether the patients with knee OA of KLG 3 or 4 underwent knee replacement surgery would have shown the importance of knee OA directly. Besides, regrettably, knee radiographs were not obtained in all of recruited patients during postoperative follow-up. Because it can be pointed out there are uncertainty that knee OA severity was aggravated from K-L grade 1 or 2 to K-L grade 3 or 4 in some patients of the group I during the postoperative follow-up. Moreover, the retrospective design introduced a degree of uncertainty due to some missing and erroneous data in medical records. In addition, as is already well known, there is little relationship between structural severity in knee joints’ pathology and clinical symptoms. Potentially, not only knee osteoarthritis but also other factors [11] including ongoing degeneration in spine itself or combined with knee issue or problem in knee only or related to muscle imbalance after surgery could affect post-surgical sagittal balance and clinical improvement. Therefore, grouping by K-L grading itself cannot represent the precise effect of osteoarthritic knee on compensatory capacity.

## Conclusions

Osteoarthritic knee with Kellgren-Lawrence Grading Scale of 3 or 4 have a negative influence on maintaining postoperative spinal sagittal alignment, balance, and clinical outcomes achieved immediately by posterior instrumentation and fusion for lumbar degenerative disease.

## Data Availability

The patients’ data were collected in Kyung Hee university hospital at Gangdong. Due to the sensitive nature of the questions asked in this study, some of survey respondents were assured raw data would remain confidential and would not be shared. (raw data unavailable).

## Abbreviations

OA: Osteoarthritis
KLG: Kellgren-Lawrence Grading
ODI: Oswestry disability index
SRS: Scoliosis Research Society
CL: Cervical lordosis
TK: Thoracic kyphosis
TLK: Thoracolumbar kyphosis
LL: Lumbar lordosis
PT: Pelvic tilt
C7SVA: C7 Sagittal vertical axis
AP: Anteroposterior
BMI: Body mass index
CrSVA-S: Cranial-sagittal vertical axis-Sacrum
CrSVA-H: Cranial-sagittal vertical axis-Hip
